# Intracranial tumors mimicking benign paroxysmal positional vertigo: A case series

**DOI:** 10.3389/fneur.2022.925883

**Published:** 2022-09-23

**Authors:** Yuan Xing Chen, Han Jun Sun, Xue Tao Mu, Chao Jiang, Hui Bing Wang, Qing Hua Zhang, Yuan Yi Qu, Jian Li, Ling Ling Zhou, Long Zhu Zhao, Ning Yu, Qing Sun

**Affiliations:** ^1^Department of Otolaryngology-Head and Neck Surgery, The Six Medical Center of Chinese PLA General Hospital, Beijing, China; ^2^Department of Otolaryngology-Head and Neck Surgery, The Third Medical Center of Chinese PLA General Hospital, Beijing, China; ^3^Department of Radiology, The Third Medical Center of Chinese PLA General Hospital, Beijing, China

**Keywords:** positional vertigo, intracranial tumor, nystagmus, mimicking, benign

## Abstract

**Background:**

A few intracranial lesions may present only with positional vertigo which are very easy to misdiagnose as benign paroxysmal positional vertigo (BPPV); the clinicians should pay more attention to this disease.

**Objectives:**

To analyze the clinical characteristics of 6 patients with intracranial tumors who only presented with positional vertigo to avoid misdiagnosing the disease.

**Material and methods:**

Six patients with intracranial tumors who only presented with positional vertigo treated in our clinic between May 2015 to May 2019 were reviewed, and the clinical symptoms, features of nystagmus, imaging presentation, and final diagnosis of the patients were evaluated.

**Results:**

All patients presented with positional vertigo and positional nystagmus induced by the changes in head position or posture, including one case with downbeating nystagmus in a positional test, two cases with left-beating nystagmus, one case with apogeotropic nystagmus in a roll test, one case with right-beating nystagmus, and one case with left-beating and upbeating nystagmus. Brain MRI showed the regions of the tumors were in the vermis of the cerebellum, the fourth ventricle, the lateral ventricle, and the cerebellar hemisphere.

Positional vertigo is a transient vertigo attack caused by gravity-related changes in head position or posture (after reaching the new head position). It can be divided into peripheral and central types. Generally, peripheral vertigo is the most common symptom (1). Benign paroxysmal positional vertigo (BPPV) is a typical peripheral vertigo, and its incidence is the highest in vertigo and dizziness diseases, accounting for 17–30% of vertigo cases (2, 3). Central paroxysmal positional vertigo (CPPV) is a position-related vertigo attack caused by a central disease. It was first described by Riesco-Macllure in 1957 (4) and is gradually concerned by more and more colleagues in neurotology and ophthalmology because it can cause serious consequences. Most patients of CPPV are accompanied by other neurological localization symptoms, which are easy to identify clinically. However, rare isolated paroxysmal positional vertigo or patients of CPPV accompanied by isolated nystagmus, are easily misdiagnosed as BPPV, and treated by repositioning maneuvers, which could delay treatment. CPPV has been reported to be related to various lesions involving the posterior fossa (such as infarction, bleeding, tumor, or demyelinating disease) (5–8). However, isolated CPPV caused only by intracranial tumors is rare. Isolated CPPV are mostly case or series reports, and there are no bulk case reports. This paper collected six patients with positional vertigo caused by intracranial tumors treated in our department from May 2015 to May 2019, focusing on the characteristics of nystagmus in position tests and the location of intracranial tumors. The report is as follows (Table 1).

**Table 1:** Clinical data of six intracranial tumor patients.

**Case**	**Sex**	**Age**	**course of disease**	**Side events/type of nystagmus/duration**			**Diseased region**	**Diagnosis**
				**DH**	**Lying down position**	**Roll test**		
1	w	27 y	2 years	Bilateral down-beating >1 min	Downbeating >1min	Bilateral downbeating >1 min	The vermis of the cerebellum	Low-grade glioma
2	w	29 y	3 months			Bilateral apogeotropic >1 min	The fourth ventricle	Medulloblastoma
3	m	54 y	1 months	Bilateral leftbeating >1 min	Leftbeating >1 min	Bilateral left-beating >1 min	The vermis of the cerebellum	Missing visits
4	m	65 y	17 years	Left Leftbeating >1min		Left Leftbeating >1min	The right lateral ventricle	Choroid plexus
5	m	53 y	2 years	Bilateral rightbeating >1 min	Rightbeating >1 min	Bilateral rightbeating >1 min	The cerebellar hemisphere and the vermicompost	hemangiobla- stomas
6	m	39 y	1 week	Left left-beating <1 min	Upbeating left torsional <1 min		The cerebellar hemisphere	Lung cancer metastasis

## Case series

### Case report 1

A 27 year old woman presented with more than 2 years history of episodic vertigo which often occurred when changing from lying position to sitting position, or from sitting position to lying position, and turning around quickly, accompanied by mild nausea. She denied any vomiting, headache, diplopia, choking cough in drinking water, dysarthria, ear symptoms such as tinnitus, ear tightness, and hearing loss, and she had clear consciousness when the episodic vertigo occurred, which often lasted for 1–2 min each time. She was initially diagnosed with BPPV in another hospital, but repositioning maneuvers for BPPV failed to relieve her symptoms. There was no progress in the disease in the past 2 years. She did not pay attention to it and did not conduct further examination. There was no spontaneous nystagmus in the examination in our hospital, and the visual eye movement, video head impulse testing, and Romberg test were normal. She had vertical downbeating nystagmus when changing from sitting position to supine position for more than 1 min in a positional test. In a roll test and Dix-Hallpike (DH) test, vertical downbeating nystagmus occurred at all positions and lasted for more than 1 min. Brain MRI showed the regions of the tumors were in the vermis of the cerebellum, which was considered as low-grade glioma, and there was no significant change in brain MRI at 8 months after diagnosis in the follow-up.

### Case report 2

**Figure 1:**
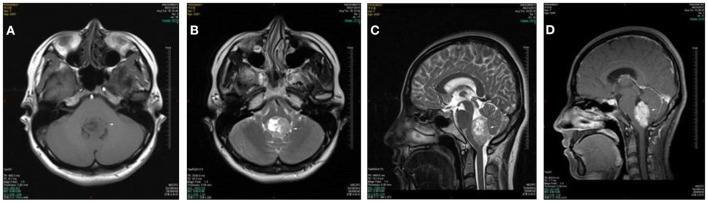
Case 2: medulloblastoma of the fourth ventricle. Axial T1WI **(A)** and Axial T2WI **(B)** show a lobulated mass in the fourth ventricle with heterogeneously iso- to hypointense signal T1WI and iso- to hyperintense signal on T2WI. The lesion extrudes posteroinferiorly through the foramen of Magendie on sagittal T2WI **(C)**. The lesion shows a moderate heterogeneous enhancement on postcontrast axial T1WI with fat saturation **(D)**.

A 29 year old woman presented with a 3-month history of positional vertigo, provoked by rolling in bed to the left and right, which was mild (Figure 1). She denied any nausea, vomiting, amaurosis fugax, diplopia, dysarthria, fear of light and sound, ear symptoms such as tinnitus, ear tightness, and hearing loss. The positional vertigo often lasted for several seconds. Her past medical history was that of migraine. She was initially diagnosed with BPPV, but repositioning maneuvers for BPPV failed to relieve her symptoms. There was no spontaneous nystagmus in the examination in our hospital, and the visual eye movement, video head impulse testing, and Romberg test were normal. The sharpened Romberg test was performed to the left. She presented with apogeotropic nystagmus induced in the roll test when rolling to left and right, which the slow phase velocity was <6°/s and lasted for more than 1 min. Brain MRI showed the regions of the tumors were in the fourth ventricle which had undergone resection and radiotherapy in another hospital. The pathological diagnosis was medulloblastoma. The positional vertigo and ambiguous speech disappeared after the operation.

### Case report 3

A 54 year old man presented with more than 1 month history of positional vertigo which often occurred when changing from lying position to sitting position or from sitting position to lying position. He denied any nausea, vomiting, headache, amaurosis fugax, diplopia, dysarthria, fear of light and sound, ear symptoms such as tinnitus, ear tightness, and hearing loss. He was relieved for several seconds and had recurrent attacks. He was initially diagnosed with BPPV in another hospital, but repositioning maneuvers for BPPV failed to relieve his symptoms. There was no spontaneous nystagmus in the examination in our hospital, and the visual eye movement, video head impulse testing, and Romberg test were normal. He had left-beating nystagmus when changing from sitting position to supine position for more than 1 min in a positional test. In a roll test and DH test, left-beating nystagmus occurred at all positions and lasted for more than 1 min. Brain MRI showed the regions of the tumors were in the vermis of the cerebellum, and metastatic tumor could not be ruled out. The patient went to another hospital for treatment without further follow-up.

### Case report 4

A 65 year old man presented with a 17-year history of positional vertigo which was often provoked by rolling in bed to the left and right, sometimes accompanied by mild headache. He denied any obvious visual rotation, nausea, vomiting, amaurosis fugax, diplopia, dysarthria, fear of light and sound, ear symptoms such as tinnitus, ear tightness, and hearing loss. He was relieved for more than 10 s. He had no significant medical history. There was no spontaneous nystagmus in the examination in our hospital, and the visual eye movement, video head impulse testing, and Romberg test were normal. He had left-beating nystagmus in the left DH test and the left roll test and lasted for more than 1 min. Brain MRI showed the regions of the tumors were in the lateral ventricle which was like a circle and had long T1 and long T2 signals. The tumor was considered as choroid plexus after the neurosurgical consultation. There was no significant change in brain MRI at seven months after diagnosis in the follow-up.

### Case report 5

**Figure 2:**
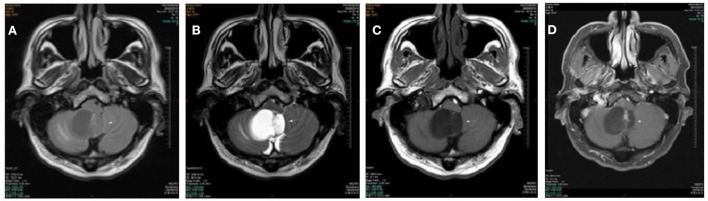
Cases 5: cerebellar hemangioblastomas. Axial T1WI **(A)**, axial T2WI **(B)**, and axial FLAIR **(C)** show a cyst mass in the cerebellar hemisphere and vermis with the marked hypointense signal on T1WI, the hyperintense signal on axial T2WI, and iso- and hypointense on FLAIR. The lesion shows septum and nodule enhancement on postcontrast axial T1WI with fat saturation **(D)**.

A 53 year old man presented with more than 2 years history of dizziness which often occurred accompanied by top-heavy and unstable walking (Figure 2). He presented with more than 3 months history of positional vertigo, worsened by rolling in bed to the left, accompanied by visual rotation and mild nausea. He denied any vomiting, headache, amaurosis fugax, diplopia, dysarthria, hearing loss, fear of light and sound. He was relieved for tens of seconds. His past medical history was that of bilateral tinnitus for more than 10 years, which did not change every time the vertigo attacked. The bilateral tinnitus was not diagnosed and treated because it was mild and had little impact on daily life. There was no spontaneous nystagmus in the examination in our hospital, and the visual eye movement, video head impulse testing, and Romberg test were normal. The sharpened Romberg test was with unstable walking. He had right-beating nystagmus when changing from sitting position to supine position for more than 1 min in a positional test. In a roll test and DH test, right-beating nystagmus occurred at all positions and lasted for more than 1 min. Brain MRI showed the regions of the tumors were in the cerebellar hemisphere and vermis, which was considered as hemangioblastomas. The patient went to another hospital for treatment without further follow-up.

### Case report 6

**Figure 3:**
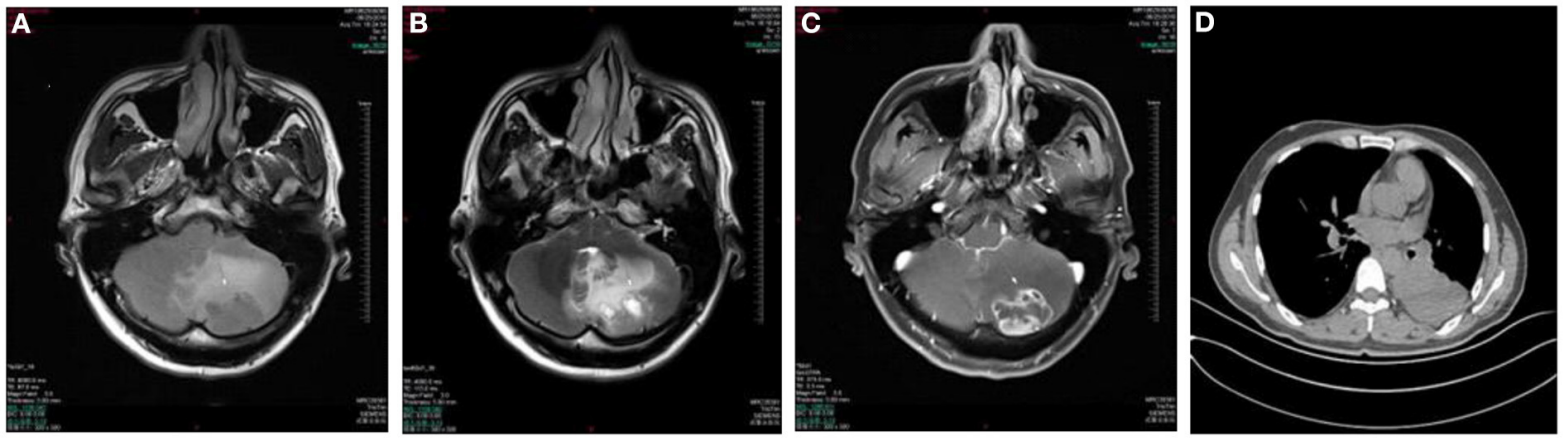
Case 6: metastasis of the left cerebellar hemisphere. Axial T1WI **(A)** and Axial T2WI **(B)** show irregular lesion and surrounding edema in the left cerebellar hemisphere with the heterogeneously hypointense signal on T1WI and marked hyperintense signal on T2WI relative to the normal parenchyma of the cerebellum. The lesion shows ring-enhancement on postcontrast axial T1WI with fat saturation **(C)**. Chest CT shows a soft tissue attenuation mass in the lower left lung **(D)**.

A 39 year old man presented with a 1-week history of episodic vertigo which was provoked by changing the head position, accompanied by mild headache and nausea (Figure 3). He denied any vomiting, amaurosis fugax, diplopia, dysarthria, hearing loss, fear of light and sound. He was relieved for several s. He was diagnosed with left lung cancer 6 months ago and underwent left lung resection in the last month. There was no spontaneous nystagmus in the examination in our hospital, and the visual eye movement, video head impulse testing, and Romberg test were normal. He had upbeating left torsional nystagmus when changing from sitting position to supine position for <1 min in positional test. He had left-beating nystagmus in the left DH test that lasted for <1 min. Brain MRI showed the regions of the tumors were in the left cerebellar hemisphere which was considered the brain metastasis of lung cancer. He was transferred to the oncology department for radiotherapy.

## Discussion

Clinically, patients with positional vertigo as the primary complaint are prevalent, most of them are BPPV; CPPV is only a minority. Intracranial tumors, often with hidden onset, can be seen in all ages. Whether the patients with an intracranial tumor only present positional vertigo depends on the region and extent of involvement of the lesion. If the lesion is small and the functional area is not involved, there may be no clinical symptoms; if the lesion is large and the other functional areas are affected, it may be accompanied by central nervous system symptoms and signs, such as double vision, facial numbness, limb numbness, dysphonia, dysphagia, etc., which is easier to identify clinically; if the lesion is relatively limited and only involves the vestibular conduction pathway, it only manifests as position-related vertigo, dizziness, and imbalance, which is easily misdiagnosed clinically. The six patients in this group showed sole positional vertigo with positional nystagmus without other central nervous system symptoms. Before the diagnosis, three patients were misdiagnosed for BPPV and were treated with repositioning maneuvers.

CPPV caused by intracranial tumors mainly violates the vestibular conduction pathway. The lesions are mostly located in the dorsal lateral part of the fourth ventricle, the dorsal part of the cerebellum, the cerebellar nodules, and the cerebellar lingual lobe (9, 10). The function of the glomus nodule is to process vertical and horizontal otolith signals. The positional nystagmus may be induced if the tumor involves the glomus nodule. However, this lesion causes vertical nystagmus and other neurological symptoms, including periodic alternating nystagmus, gaze induced nystagmus, and ataxia. Besides the semicircular canal, the cerebellar nodules and the cerebellar lingual lobe are also connected with otolith organs, which control the regulation of vestibular ocular reflex. If the lesion only involves the cerebellar nodules and the cerebellar lingual lobe, it does not cause other neurological symptoms, and the nystagmus caused is not only vertical (8, 11, 12). The lesion regions of the six patients in this group, except for case 2 whose lesion was located in the lateral ventricle, were common sites with CPPV symptom, including one case in the fourth ventricle, two cases in the cerebellar vermis, and two cases in both the cerebellar hemisphere and the cerebellar vermis. The tumors in the lateral ventricle theoretically do not cause positional nystagmus, and the patient had no significant medical history and there were no other lesions in the examination in our hospital. However, the same nystagmus was induced in positional tests during the past three times. The positional nystagmus may not be caused by direct stimulation of the vestibular system by tumor. During head position change, the flow of cerebrospinal fluid is blocked by the mass and reflux, indirectly stimulating the vestibular center and causing positional vertigo. However, the follow-up needs to continue to track whether the patient has new lesions. Common pathological types of intracranial tumors inducing CPPV include neurogenic tumors, meningiomas, gliomas, metastases, etc. There was one case of low-grade glioma, one case of medulloblastoma, one case of choroid plexus, one case of hemangioblastomas, one case of lung cancer metastasis and one case of lost follow-up in the group. There was no number of cases of one pathological type being the majority.

Since the clinical incidence of CPPV is low, Cranial CT or craniocerebral MRI is not a routine examination for patients with positional vertigo. If patients do not present with central nervous system symptoms, nystagmus in positional tests is often the first breakthrough. Central paroxysmal positional nystagmus (CPPN) associated with central lesions is often different from the nystagmus in BPPV and it has its independent characteristics. CPPN usually has no latency, can appear in multiple positions, has no noticeable change in strength, and has no attenuation in the fixation test. CPPN cannot be explained by the theory of canalith flow (9, 13, 14). In 2017 (15), Macdonald summarized the nystagmus characteristics of 82 CPPV patients in 28 articles. The results are as follows: (1) CPPV patients often have nystagmus in the DH test, roll test, head suspension test, and other examinations; (2) Among the patients with positive DH test, about 60% are positive on both sides, of which 47.5% are vertical nystagmus; (3) Most of the nystagmus induced by the DH test and head suspension test lasts for <30 s, roll test Induced nystagmus lasts for <1 min for geotropic nystagmus and >1 min for apogeotropic nystagmus; (4) Most CPPN has no latency, fatigue, and fixation suppression disappears. In our six cases, the head suspension test was not performed, and the nystagmus in the supine position was observed in the roll test. The summary is as follows: (1) Case 1, 3, and 5 induced the nystagmus in the same direction at all positions. Case 1 was vertical downbeating nystagmus. Case 3 and 5 were horizontal-beating nystagmus. Case 4 had left-beating nystagmus in the left DH test and the left roll test. Case 2 presented with apogeotropic nystagmus induced in a roll test. Case 6 had upbeating left torsional nystagmus when changing from sitting position to supine position in positional test and left-beating nystagmus in the left DH test. (2) Case 2 had apogeotropic direction-changing positional nystagmus induced by the roll test and was treated with canalith repositioning procedures (CRP). Nystagmus analysis showed the slow phase velocity of the bilateral nystagmus in the roll test was <6°/s, which was not consistent with the characteristic of nystagmus in BPPV, as in the other five cases. (3) The duration of nystagmus in case 6 was <1 min, the other five cases were more than 1 min, and it had no characteristics of becoming stronger and weaker. The analysis is as follows: (1) All patients showed induced nystagmus in at least two positions, and in four of the patients, nystagmus did not change with positional changes. The reason may be that the lesion was located in the center and there was no flow of semicircular canal stones in patients with BPPV after postural change; it was only manifested as the asymmetry of sensory signals in bilateral vestibular center, which induced specific nystagmus through specific conduction pathways. (2) The duration of nystagmus in five patients was more than 1 min, and there was no visible fatigue, which was not consistent with the characteristics of nystagmus described by Macdonald, but was consistent with the characteristics of central nystagmus. (3) Only one patient showed the induced vertical nystagmus, one patient had the induced torsional nystagmus at one position, and four patients had horizontal nystagmus. Vertical nystagmus is not a common type in our cases. It may be because of the small number of cases in our study. (4) One patient had apogeotropic direction-changing positional nystagmus induced by the roll test and the slow phase velocity of the bilateral nystagmus was <6°/s. The nystagmus on one side was not significantly stronger than the nystagmus on the other side, which was not consistent with the characteristic of nystagmus in classical BPPV.

CPPV needs to be differentiated from other diseases, first of which is BPPV. For most patients with BPPV, the characteristics of nystagmus are consistent with the canalith theory, and the treatment effect is good. Next is the relatively rare cupulolithiasis of the horizontal semicircular canal BPPV. Its nystagmus often has no incubation period, easy fatigue, and has a great duration (16), in addition, the effect of CRP is relatively weak. In particular, clinicians should avoid misdiagnosis and missed diagnosis. In vestibular migraine (VM), most VM patients can present with positional vertigo during the onset period, about 1% of VM patients show isolated positional vertigo (7) because it is a central nervous system disease. Its nystagmus can also show the characteristics of central nystagmus, which may be confused with CPPV, but VM is often accompanied by symptoms such as headache, photophobia, phobia, and migraine characteristics such as family history and motion sickness (17), which can help identify the correct diagnosis. For vestibular paroxysmia (VP), which is caused by the compression of the eighth cranial nerve, rare in the clinic, and can be manifested as stereotyped and positional vertigo. The experimental treatment with carbamazepine is effective for collaborative diagnosis, and brain MRI is used to differentiate VP from the intracranial tumor (18).

Although CPPV is rare in the clinic, once it is a missed diagnosis, it may cause serious consequences. When patients complain of positional vertigo, nystagmus characteristics are inconsistent with BPPV, and CRP is invalid, etc. we should think of the possibility of CPPV, take a craniocerebral MRI examination promptly, determine if there are intracranial lesions, and get timely and appropriate treatments.

## Conclusions and significance

When presenting with nystagmus features that are not consistent with BPPV, patients should receive a brain MRI examination to distinguish BPPV from intracranial diseases.

## Data availability statement

The original contributions presented in the study are included in the article/supplementary material, further inquiries can be directed to the corresponding author/s.

## Ethics statement

The studies involving human participants were reviewed and approved by the Ethics Committee of the Chinese PLA General Hospital (S2020-465-01). The patients/participants provided their written informed consent to participate in this study. Written informed consent was obtained from the individual(s) for the publication of any potentially identifiable images or data included in this article.

## Author contributions

QS and NY designed the experimental paradigm, summarized the results, and wrote the article. YC collected the clinical cases. HS, XM, CJ, HW, QZ, YQ, JL, LLZ, and LZZ examined the patients, like hearing examination, vestibular function examination, and imaging examination. All authors contributed to the article and approved the submitted version.

## Funding

This research was supported by Beijing Natural Science Foundation (7222185), National Foundation (2020YFC2004001), and NSFC (81470700).

## Conflict of interest

The authors declare that the research was conducted in the absence of any commercial or financial relationships that could be construed as a potential conflict of interest.

## Publisher's note

All claims expressed in this article are solely those of the authors and do not necessarily represent those of their affiliated organizations, or those of the publisher, the editors and the reviewers. Any product that may be evaluated in this article, or claim that may be made by its manufacturer, is not guaranteed or endorsed by the publisher.
